# The Effect of Bioactive Compounds on *In Vitro* and *In Vivo* Antioxidant Activity of Different Berry Juices

**DOI:** 10.1371/journal.pone.0047880

**Published:** 2012-10-23

**Authors:** Ana Slatnar, Jerneja Jakopic, Franci Stampar, Robert Veberic, Polona Jamnik

**Affiliations:** 1 Biotehnical Faculty, Agronomy Department, Jamnikarjeva, Ljubljana, Slovenia; 2 Biotehnical Faculty, Department of Food Science and Technology, Jamnikarjeva, Ljubljana, Slovenia; University of Sassari, Italy

## Abstract

**Background:**

Berry fruit is known for its high contents of various bioactive compounds. The latter constitute of anthocyanins, flavonols and flavanols and posses high antioxidative activity. The highly dynamic antioxidant system can be evaluated *in vitro* and *in vivo* in several model organisms. These measurements represent a good approximation of the real potential of bioactive compounds in the cells of higher eucarions. The aim of the study was thus to determine *in vitro* and *in vivo* antioxidant activity of different berry juices, which reportedly contain high amounts of phenolics.

**Methodology/Principal Findings:**

Five different berry species were collected from several locations in central Slovenia and juice was extracted from each species separately. Juice was assessed for their *in vitro* and *in vivo* antioxidant activity. Phenolic profiles of berries were determined with the use of a HPLC/MS system, *in vitro* antioxidant activity with the DPPH radical scavenging method and *in vivo* antioxidative activity using *Saccharomyces cerevisiae.* The highest diversity of individual phenols was detected for bilberry juice. The highest *in vitro* antioxidant capacity was determined for blackcurrant juice. A decrease in intracellular oxidation compared to control was observed in the following order: blackcurrant < chokeberry = blueberry < bilberry. The results indicate important differences in antioxidant activity of berry juices between *in vitro* and *in vivo* studies.

**Conclusion/Significance:**

In addition to the total content of phenolic compounds entering the cells, a key factor determining antioxidative activity of berry juices is also the ratio between the compounds. Where high content levels of anthocyanins and very low content levels of flavonols and hydroxycinnamic acids were measured a lower intracellular oxidation has been detected. Specifically, intracellular oxidation increased with higher consumption of hydroxycinnamic acids and lower consumption of anthocyanins in the cells. Antioxidative activity also increased when the consumption of analyzed phenols was rather low.

## Introduction

Berry fruits are characterized by a high content and wide diversity of phenolic compounds. They differ in their structure and molecular weight and are frequently represented by phenolic acids (benzoic and cinnamic acid derivatives), tannins, stilbenes and flavonoids such as anthocyanidins, flavonols and flavanols [1], [2]. Their concentration is usually higher in the epidermis and in the tissue directly under the skin compared to the central part of the fruit. Among different bioactive substances detected in berries, phenolic compounds such as flavonoids, tannins and phenolic acids stand out and exhibit a wide range of biological effects, including antioxidant [3], [4] and anticarcinogenic properties. Epidemiological evidence suggests that high consumption of flavonoids may provide protection against coronary heart disease [5], stroke and lung cancer [6].

Fruit extracts, containing phenolic compounds, are characterized by higher antioxidant activity than many isolated pure phenolic compounds or vitamins [7]. This points out to the synergistic effect of antioxidants and selected phenolics in the extract. According to Wang et al. [8], ascorbic acid activity is similar to Trolox activity, while the activity of some polyphenols is several fold higher than the reference standards. For example, antioxidant activity of cyanidin is 4.4 fold higher compared to ascorbic acid, whereas quercetin and tannins exhibited as much as 4.7 and 3 to 30 fold higher activity, respectively.

These phenolic compounds potentially inhibit cell damage caused by oxidation, which can lead to several pathological conditions [9]. However, on the basis of *in vitro* studies, antioxidant activity of specific bioactive compounds can only partly be correlated to the measured antioxidant activity in the cells. Therefore, cellular antioxidant activity assay was developed to precisely measure the antioxidant activity of selected antioxidants, dietary supplements, and foods in *in vivo* cell culture [10]. Also, a better understanding of phenolic pharmacodynamics is crucial for accurate antioxidative evaluation. Therefore, many studies are being carried out on the molecular and genetic interactions of phytochemicals and other bioactive substances in food and dietary supplements [11].

Yeast *Saccharomyces cerevisiae* is an appropriate model organism for fundamental eukaryotic cellular processes such as cell antioxidative activity related to bioactive compounds [11]–[14]. Since oxidative damages of proteins, lipids, nucleic acids and other cell components as well as defence systems against oxidative stress (e.g., the nature and role of enzymes participating in decreasing reactive oxygen species level (ROS), repair of damaged macromolecules (primarily DNA) and elimination of irreparable proteins) are basically almost similar at all levels of cellular organization, microorganisms are useful models for studying various aspects of oxidative stress at the biochemical, molecular biological and cellular levels [15].

To our knowledge, none of the *in vivo* studies have investigated the relationships between antioxidative activity and phenolic composition of various berry juices in a single experiment. The aim of the study was the determination of *in vitro* and *in vivo* antioxidant activity of different berry juices, which reportedly contain high amounts of phenolics. The present investigation also focused on expanding the existing initial component analysis by using high performance liquid chromatography (HPLC) equipped with a photodiode array detector (PAD) and mass spectrometer (MS) to elucidate any differences in the content and profile of selected flavonoid and phenolic acids present in different berry juices. Additionally, the uptake of these compounds was measured in the yeast cells. In literature, various methods have often been used in order to analyse the antioxidant potential of dietary substances and these assays could present the basis for further studies on antioxidant activity of specific bioactive compounds in the human body.

## Materials and Methods

### Ethics Statement

No specific permits were required for the described field study. The location was not privately-owned or protected in any way so the plant material can freely be harvested by anyone.

### Plant Material and Juice Preparation

Five different berry species: elderberry (*Sambucus nigra*), blackcurrant (*Ribes nigrum*), blueberry (*Vaccinium corymbosum*), chokeberry (*Aronia melanocarpa*) and bilberry (*Vaccinium myrtillus*), were collected from several locations in central Slovenia (latitude: 46° 2′, longitude: 14° 28′). Fruit samples were harvested from July to September 2011 at full maturity. Only undamaged fruit were selected, and juice was prepared from 0.5 kg of each species by squeezing berries in a mortar. The obtained juice was then centrifuged for 10 min at 12,000 g_n_. The supernatant was filtered through a Chromafil AO-25/25 polyamide filter (Macherey-Nagel) and transferred to a vial prior to injection into a HPLC system, determination of antioxidant activity with the DPPH radical scavenging method and determination of intracellular oxidation.

### Determination of Individual Phenolic Compounds

Samples were analyzed using a Thermo Finnigan Surveyor HPLC system (Thermo Scientific, San Jose, CA) with a PAD at 280 nm (hydroxycinnamic acids), 350 nm (flavonols) and 530 nm (anthocyanins). A Phenomenex (Torrance, CA) HPLC column C18 (150×4.6 mm, Gemini 3µ) protected with a Phenomenex security guard column operated at 25°C was used. The injection volume was 20 µL and the flow rate maintained at 1 mL min^−1^. The elution solvents were aqueous 1% formic acid and 5% acetonitrile (A) and 100% acetonitrile (B). Samples were eluted according to the gradient described by Marks, Mullen, & Crozier [16]: 0–5 min, 3% to 9% B; 5–15 min, 9% to 16% B; 15–45 min, 16% to 50% B; 45–50 min, 50% isocratic; and finally washing and reconditioning of the column. Identification of compounds was achieved by comparing retention times and their UV-VIS spectra from 200 to 600 nm, as well as by the addition of an external standard. Compounds were identified using a mass spectrometer (Thermo Scientific, LCQ Deca XP MAX) with an electrospray ionisation (ESI) operating in negative/positive ion mode. The analyses were carried out using full-scan data dependent MS^n^ scanning from *m/z* 115 to 2000. The capillary temperature was 250°C, the sheath gas and auxiliary gas were 20 and 7 units, respectively and the source voltage was 4 kV for negative ionisation and 0.1 kV for positive ionisation.

Concentrations of phenolic compounds were calculated from peak areas of the sample and the corresponding standards. The concentrations were expressed in µg mL^−1^.

### Determination of Antioxidant Activity with the DPPH Radical Scavenging Method

The juice of fruit samples for the determination of antioxidant activity was made according to the same protocol as for individual phenolics. The free radical scavenging activity of different berry juices was measured according to the DPPH method reported by Brand-Williams, Cuvelier and Berset [17] with some modifications. Berry juice samples (50 µL) were placed in 96-well microplates, and 200 µL of 0.1 mmol L^−1^ methanolic solution of DPPH was added and allowed to react in the dark at room temperature. The decrease of DPPH absorbance was measured at 520 nm at 5 min intervals by a spectrophotometer (MRX Dynex Technologies), until absorbance stabilized (30 min). Water was used as blank solution, and DPPH solution without test samples served as the control. All sample analyses were performed in triplicate. The DPPH radical scavenging activity of different berry juices was expressed as % of reacted DPPH in 30 min of reaction time.

### Yeast Strain and Cultivation

The yeast *S. cerevisiae* ZIM 2155 was obtained from the Culture Collection of Industrial Microorganisms (ZIM) of the Biotechnical Faculty, University of Ljubljana, Ljubljana, Slovenia.

The yeast cells were cultivated in a yeast extract (10 g L^−1^; Biolife), peptone (20 g L^−1^; Biolife), glucose (20 g L^−1^; Merck) (YEPD) medium at 28°C and 220 rpm, until their stationary phase was achieved. The cells were then centrifuged for 3 min at 4,000 *g_n_*, washed once with phosphate-buffered saline (PBS) (Merck) and suspended in PBS at a concentration of 1×10^8^ cells mL^−1^. The cells were further incubated at 28°C and 220 rpm, for 96 h.

### Treatment of Yeast Cells

The juice of 5 different fruit samples was added to the yeast cell suspensions following their 96-h incubation in PBS, at different concentrations (1, 2.5, 5 and 10 µL juice mL^−1^ of yeast cell suspension).

After further 2-h incubation at 28°C and 220 rpm, the samples were taken for further analyses: measurements of intracellular oxidation, cell viability and cellular uptake.

### Determination of Cell Viability

Cell viability was measured as CFU (colony forming units). After 2-h incubation CFU were determined by plating cell suspension on yeast extract (10 g L^−1^; Biolife), peptone (20 g L^−1^; Biolife), glucose (20 g L^−1^; Merck), agar (20 g L^−1^; Biolife) (YEPD) medium and then after a 2-day incubation at 28°C the number of colonies was counted. The experiments were performed in biological duplicates. Data are expressed as means of CFU mL^−1^± SE.

### Determination of Intracellular Oxidation

Intracellular oxidation was estimated using 2`,7`-dichlorofluorescein (H_2_DCF), which reacts with oxidants, thus revealing the presence of ROS. This was given to the cells as 2`,7`-dichlorofluorescein diacetate (H_2_DCFDA), which easily penetrates the plasma membrane and is hydrolysed inside the cells by non-specific esterases. The non-fluorescent H_2_DCF can then be oxidised to fluorescent 2`,7`-dichlorofluorescin (DCF), which is measured fluorimetrically [18].

The cells from the 2-ml incubations were sedimented by centrifugation (14,000 *g_n_*, 5 min), and washed three times with 50 mM potassium phosphate buffer (pH 7.8). The cell pellets were finally resuspended in 9 volumes of 50 mM potassium phosphate buffer (to 10%, v/v) and incubated at 28°C for 5 min. The ROS-sensing dye H_2_DCFDA (Sigma) was added from a 1 mM stock solution in 96% ethanol (Merck), to a final concentration of 10 µM. After 20-min incubation at 28°C and 220 rpm, the fluorescence of yeast cell suspension was measured in a kinetic mode, using a Safire II microplate reader (Tecan). The excitation and emission wavelengths of DCF were 488 nm and 520 nm, respectively. Values of fluorescence intensity were measured at 90 min. Data are expressed as means of the relative fluorescence intensity ± SE.

### Determination of Cellular Uptake

For the analysis of cellular uptake the yeast cell suspension before and after exposure to berry juices was centrifuged at 14,000 *g_n_* for 5 min). The supernatants were filtered (pore size: 0.2 µm) and analysed as described in the section reporting the determination of individual phenolic compounds.

### Chemicals

The following standards were used for the quantification of phenolic compounds: chlorogenic acid (3-caffeoylquinic acid), rutin (quercetin-3-*O*-rutinoside), cyanidin-3-*O*-galactoside from Sigma-Aldrich, quercetin-3-*O*-glucoside, quercetin-3-*O*-galactoside from Fluka Chemie (Buchs, Switzerland). The chemicals for the mobile phases were HPLC-MS grade acetonitrile and formic acid from Fluka Chemie. Water for the mobile phase was twice distilled and purified with the Milli-Q system (Millipore, Bedford, MA). For the determination of antioxidant capacity 1,1-diphenyl-2-picrylhydrazyl (DPPH) was purchased from Sigma.

### Statistical Analysis

The experiments were performed in biological duplicates and within each duplicate three technical replicates were done. Data are expressed as means of the relative fluorescence intensity or % reacted DPPH ± SE. The data was analyzed using the Statgraphics Plus 4.0 program (Manugistics. Inc.; Rockville, Maryland, USA). Differences in antioxidant activity between berry juices were analyzed with one-way analysis of variance (ANOVA). Significant differences among means were determined by Duncan’s Multiple Range test with a significance level of 0.05.

## Results and Discussion

In all juice samples 41 different bioactive compounds were identified and classified to the following phenolic groups: hydroxycinnamic acids, flavonols and anthocyanins. The highest diversity of individual phenols was found in bilberry juice, where a total of 19 compounds were determined. The second largest diversity of phenolic compounds was measured in blueberry juice (17 compounds), followed by blackcurrant juice (15 compounds) and chokeberry juice (12 compounds). The smallest structural variability was found in elderberry juice.

The differences in phenolic diversity of berry juices is to be expected as several authors report significant differences in phenolic profiles of berry species. The concentration of hydroxycinnamic acids ranged from 260.1 to 2048.2 µg mL^−1^, representing from 3.2 to 40.4% total analysed phenolic compounds in different berry juices. Flavonols were the most diverse phenolic group in berry juices, but their concentration was relatively low and only represented from 1.1 to 19.2% of total analyzed phenolic compounds (40.3 to 363.6 µg mL^−1^). Anthocyanins were the most abundant phenolics in berry juices representing from 40.4 to 92.3% of total analyzed compounds (539.6 to 7440.9 µg mL^−1^) (Table 1). The heterogeneous juice composition is a good indicator of all further differences in the study.

**Table 1:** Content levels of phenolic compounds in berry juice (mean, µg mL^−1^).

	elderberry	blackcurrant	blueberry	chokeberry	bilberry
**Hydroxycinnamic acids**	260.13	332.47	539.62	2048.23	258.10
**Total flavonols**	363.61	258.29	255.60	78.51	40.30
quercetin glycosides^a^	361.31	50.57	177.99	78.51	20.46
kaempferol glycosides	2.29	55.85	5.32	/	/
myricetin glycosides	/	151.79	/	/	13.93
ishoramnethin glycosides	/	/	47.89	/	2.23
syringetin glycosides	/	/	24.39	/	3.73
**Total anthocyanidins**	7440.85	7110.65	539.62	3569.00	3269.39
cyanidin glycosides	7440.85	2558.58	402.83	3569.00	1340.56
delfinidin glycosides	/	4438.93	/	/	1137.90
petunidin glycosides	/	78.68	/	/	/
peonidin glycosides	/	34.51	/	/	406.83
malvidin glycosides	/	/	49.74	/	384.10
unknown	/	/	87.05	/	/

a sum of different glycosides.

Based on the reports of several studies [19]–[21], berries, as well as berry juices included in our study possess a high *in vitro* antioxidant potential due to their high phenolic levels. In our experiment, antioxidant capacity was measured according to the DPPH method and indicated statistical differences between different berry juices. The highest antioxidant capacity was determined in blackcurrant juice, followed by elderberry, chokeberry, bilberry and blueberry juices. Additionally, statistical differences were not observed between elderberry and chokeberry juice antioxidant capacity (Table 2).

**Table 2:** Antioxidant capacity of berry juice (DPPH method).

species	% DPPH		
elderberry	60.87	±4.43	b
blackcurrant	73.55	±2.13	a
blueberry	8.48	±0.88	d
chokeberry	58.94	±3.79	b
bilberry	43.17	±1.99	c

Data are the mean ± SE Values described with different letters (a-d) are significantly different (Duncan's multiple-range test; p<0.05).

**Figure 1:**
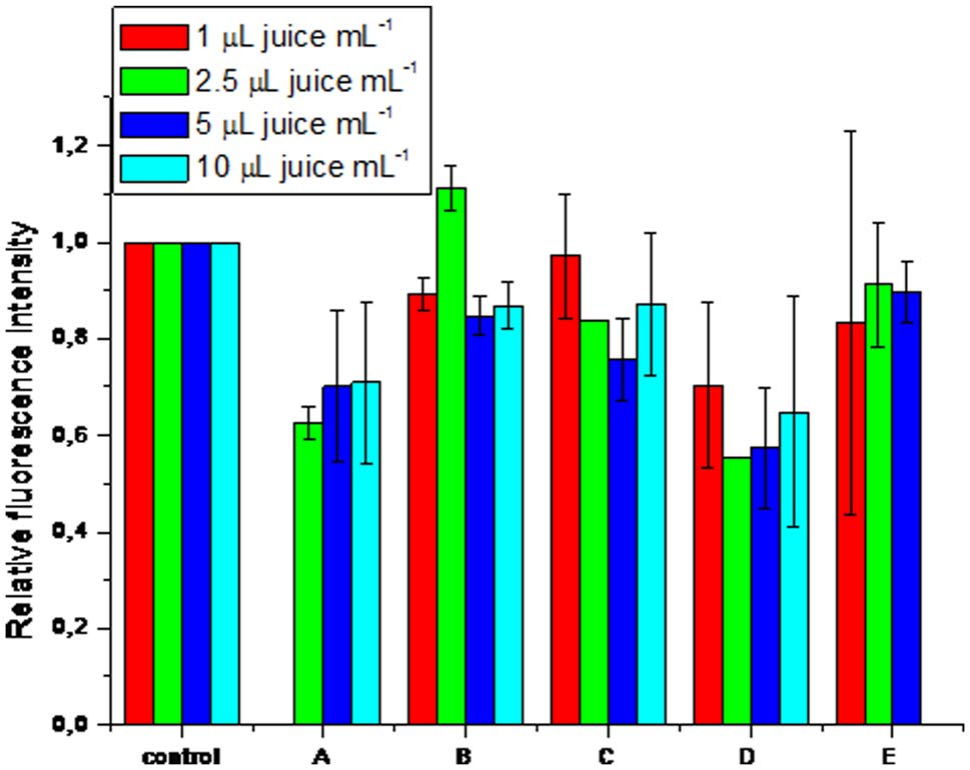
Intracellular oxidation in the yeast *S. cerevisiae* after 2-h exposure to different berry juices at different concentrations.

**Figure 2:**
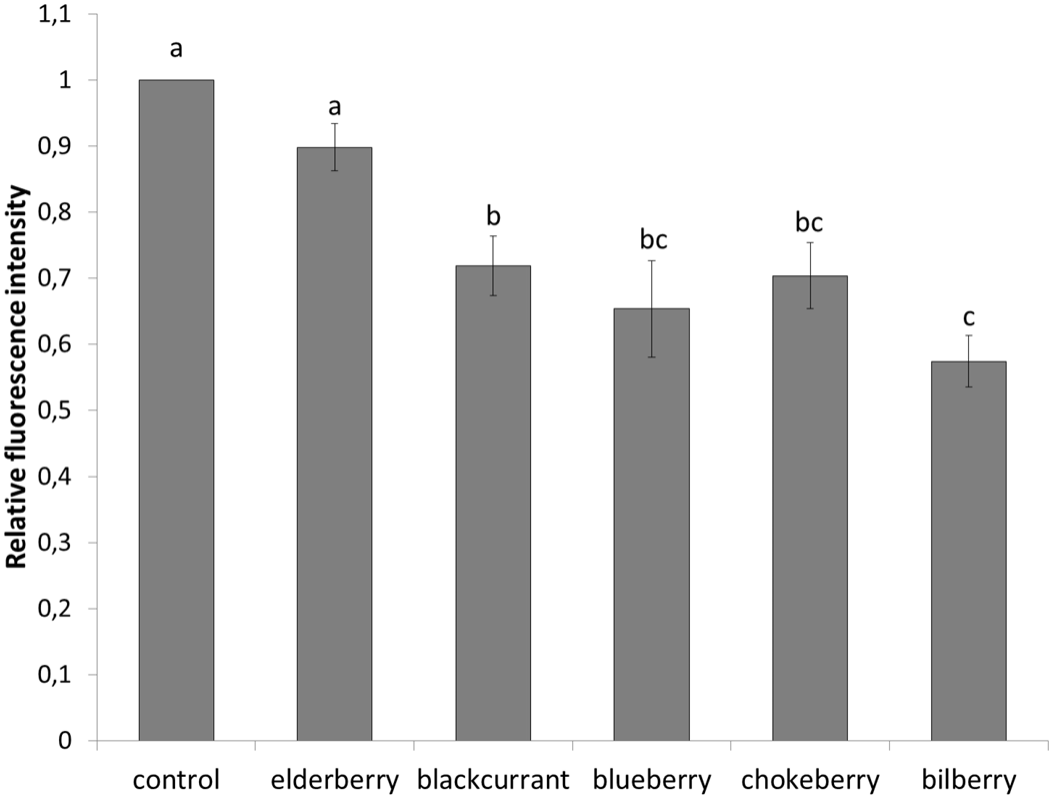
Intracellular oxidation in the yeast *S. cerevisiae* after 2-h exposure to different berry juices. Data are the mean ± SE, with values not sharing a common letter (a-c) being significantly different (Duncan's multiple-range test; p<0.05).

However, antioxidant activity of bioactive compounds in the cell cannot merely be predicted on the basis of *in vitro* studies of the antioxidative capacity of berry juices. Therefore, the effect of different berry juices on the oxidant level in the cell was investigated using yeast *Saccharomyces cerevisiae* as a model organism. Yeast cells were treated for 2 h with different berry juices at several concentrations (1, 2.5, 5 and 10 µL juice mL^−1^ of yeast cell suspension). Results did not show statistically significant differences in the intracellular oxidation between the concentrations used (Figure 1). Based on these measurements, the concentration of 5 µL juice mL^−1^ of yeast cell suspension was used for further studies. Elderberry juice sample did not show statistical differences in intracellular oxidation compared to the control treatment. A decrease in intracellular oxidation compared to the control was observed in the following order: blackcurrant < chokeberry = blueberry < bilberry (Figure 2).

**Figure 3:**
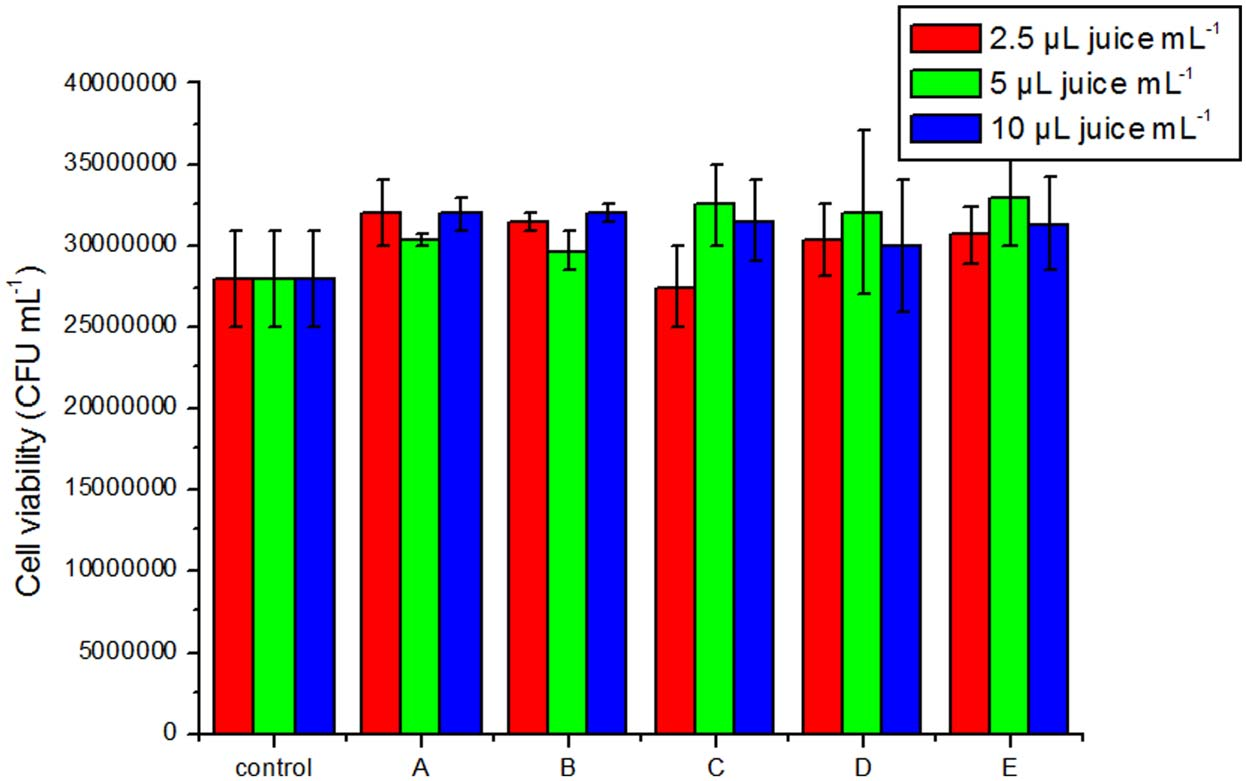
Cell viability of yeast *S. cerevisiae* exposed to different concentrations of juices fo 2 h. A – chockeberry, B – blackcurrant, C – blueberry, D – bilberry, E – elderberry. Data are expressed as means of CFU/mL ±SE.

Additionally, all five juices were tested at different concentrations for potencial toxicity in model organism by determining cell viability measured as CFU. Results showed that none of juices at different concentrations did not show a decrease in cell viability compared to the control (Figure 3).

The results concerning antioxidant activity of berry juices indicate differences between *in vitro* and *in vivo* studies. To better understand which bioactive compounds are responsible for antioxidative activity in the cell, the content of phenolic compounds was measured before and after exposure of yeast cells to different berry juices.

In the group of hydroxycinnamic acids three compounds were identified. Chlorogenic acid was determined in all analysed berry juices. On the other hand, cryptochlorogenic acid was only detected in bilberry juice, and caffeoylhexose in blackcurrant juice. The content of hydroxycinnamic acids in yeast cell suspension are presented in Table 3. The relative percentage of hydroxycinnamic acids was highest in blueberry (63%) and chokeberry (36%) juice, and their content was from 8 to 28 fold higher compared to bilberry, elderberry and blackcurrant juice.

**Table 3:** Analyzed phenolic groups of different berry juices and their content levels in yeast cell suspension (mean, µg mL^−1^).

	elderberry		blackcurrant		blueberry		chokeberry		bilberry	
	before	after	before	after	before	after	before	after	before	after
**Hydroxycinnamic acids**	**1.30**	**0.81**	**1.66**	**0.78**	**6.88**	**0.00**	**10.24**	**7.12**	**1.29**	**0.00**
**Total flavonols**	**1.82**	**1.37**	**1.29**	**0.92**	**1.28**	**0.00**	**0.39**	**0.28**	**0.21**	**0.00**
quercetin glycosides^a^	1.81	1.36	0.25	0.21	0.90	0.00	0.39	0.31	0.10	0.00
kaempferol glycosides	0.01	0.01	0.29	0.19	0.03	0.00	/	/	/	/
myricetin glycosides	/	/	0.76	0.52	/	/	/	/	0.07	0.00
ishoramnethin glycosides	/	/	/	/	0.24	0.00	/	/	0.01	0.00
syringetin glycosides	/	/	/	/	0.12	0.00	/	/	0.01	0.00
**Total anthocyanidins**	**37.20**	**6.47**	**35.55**	**12.59**	**2.70**	**0.00**	**17.84**	**1.91**	**16.35**	**0.00**
cyanidin glycosides	37.20	6.47	12.79	5.31	2.01	0.00	17.84	1.91	6.70	0.00
delfinidin glycosides	/	/	22.20	7.28	/	/	/	/	5.69	0.00
petunidin glycosides	/	/	0.39	0.00	/	/	/	/	/	/
peonidin glycosides	/	/	0.17	0.00	/	/	/	/	2.03	0.00
malvidin glycosides	/	/	/	/	0.25	0.00	/	/	1.92	0.00
unknown	/	/	/	/	0.43	0.00	/	/	/	/

a sum of different glycosides.

The group of flavonols stands out in its phenolic diversity. Twenty (20) different compounds were identified from this group; the largest subgroup comprised of quercetin-glycosides, but several myricetin, kaempferol, ishoramnetin and syringetin glycosides were also determined. Even though flavonols were the most heterogeneous group identified in berry juices their content only represented from 1 to 12% total analysed phenols. The smallest flavonol diversity was determined in elderberry juice, but oppositely, their content was highest. On the other hand, the largest variability of flavonols was established for blueberry and bilberry juices, but the concentration of individual compounds was very low in blueberry juice.

A high concentration of anthocyanins has previously been reported in chokeberry, bilberry, blackcurrant, and elderberry fruit [21]–[24]. Anthocyanins comprise a large group of water-soluble pigments and are found mainly in the external layers of the fruit hypodermis (the skin). In plant cells, they are present in vacuoles in the form of various sized granules. Oppositely, cell walls and flesh tissue contain practically no anthocyanins, with the exception of bilberry fruit, which also contains lower levels of anthocyanins in the flesh. In berry juices 17 anthocyanins were determined and identified as glycosides of cyanidin, delphinidin, petunidin, peonidin and malvidin. Anthocyanins represented between 25 and 92% of all analysed phenolics in different berry juices. Cyanidin and delphinidin glycosides were detected in highest concentrations, and glycosides of other aglycones in lower concentrations (Table 3).

The results of cellular uptake indicate that the analysed compounds all entered the yeast cells, but their total uptake was different and ranged from 62.9 and 100%. Additionally, yeast cell phenolic uptake was also quantified and ranged from 108.6 to 316.7 µg. These points out that the phenolic uptake of the yeast cells was 2.9 fold lower compared to the available compounds in cell suspension. Interestingly, an increased phenolic uptake did not trigger a higher *in vivo* antioxidant activity. This was demonstrated for bilberry and blueberry juices, where the highest levels of antioxidant activity (Figure 2) and simultaneously the lowest phenolic uptake have been measured (Table 4).

**Table 4:** Uptake of different phenolic groups (mean in µg and %).

	elderberryuptake		blackcurrantuptake		blueberryuptake		chokeberryuptake		bilberryuptake	
	µg	%	µg	%	µg	%	µg	%	µg	%
**Hydroxycinnamic acids**	**4.9**	**37.7**	**8.8**	**53**	**68.8**	**100**	**31.2**	**30.5**	**12.9**	**100**
**Total flavonols**	**4.5**	**24.7**	**3.7**	**28.7**	**12.8**	**100**	**1.1**	**28.2**	**2.1**	**100**
quercetin glycosides^a^	4.5	24.8	0.4	16	9.0	100	0.8	20.5	1.0	100
kaempferol glycosides	0	100	1.0	34.5		100	/	/	/	/
myricetin glycosides	/	/	2.4	21.6	/	/	/	/	0.7	100
ishoramnethin glycosides	/	/	/	/	2.4	100	/	/	0.1	100
syringetin glycosides	/	/	/	/	1.2	100	/	/	0.1	100
**Total anthocyanidins**	**307.3**	**82.6**	**229.6**	**64.6**	**27.0**	**100**	**159.3**	**89.3**	**163.5**	**100**
cyanidin glycosides	307.3	82.6	74.8	48.5	20.1	100	159.3	89.2	67.0	100
delfinidin glycosides	/	/	149.2	42.2	/	/	/	/	56.9	100
petunidin glycosides	/	/	3.9	100	/	/	/	/	/	/
peonidin glycosides	/	/	1.7	100	/	/	/	/	20.3	100
malvidin glycosides	/	/	/	/	2.5	100	/	/	19.2	100
unknown	/	/	/	/	4.3	100	/	/	/	/
**Total**	**316.7**	**78.5**	**242.1**	**62.9**	**108.6**	**100**	**191.6**	**67.3**	**178.5**	**100**

a sum of different glycosides.

The total hydroxycinnamic acids uptake of analysed berry juices ranged between 30.5 and 100% (from 4.9 to 68.8 µg). The highest hydroxycinnamic acids uptake was measured in blueberry and bilberry juices, and the lowest in chokeberry juice. The content of consumed flavonols ranged between 24.7 and 100%, which represents from 1.1 to 12.8 µg flavonol uptake of the cells. The anthocyanin uptake was between 64.6–100% (from 27.0 to 307.3 µg) (Table 4).

**Figure 4:**
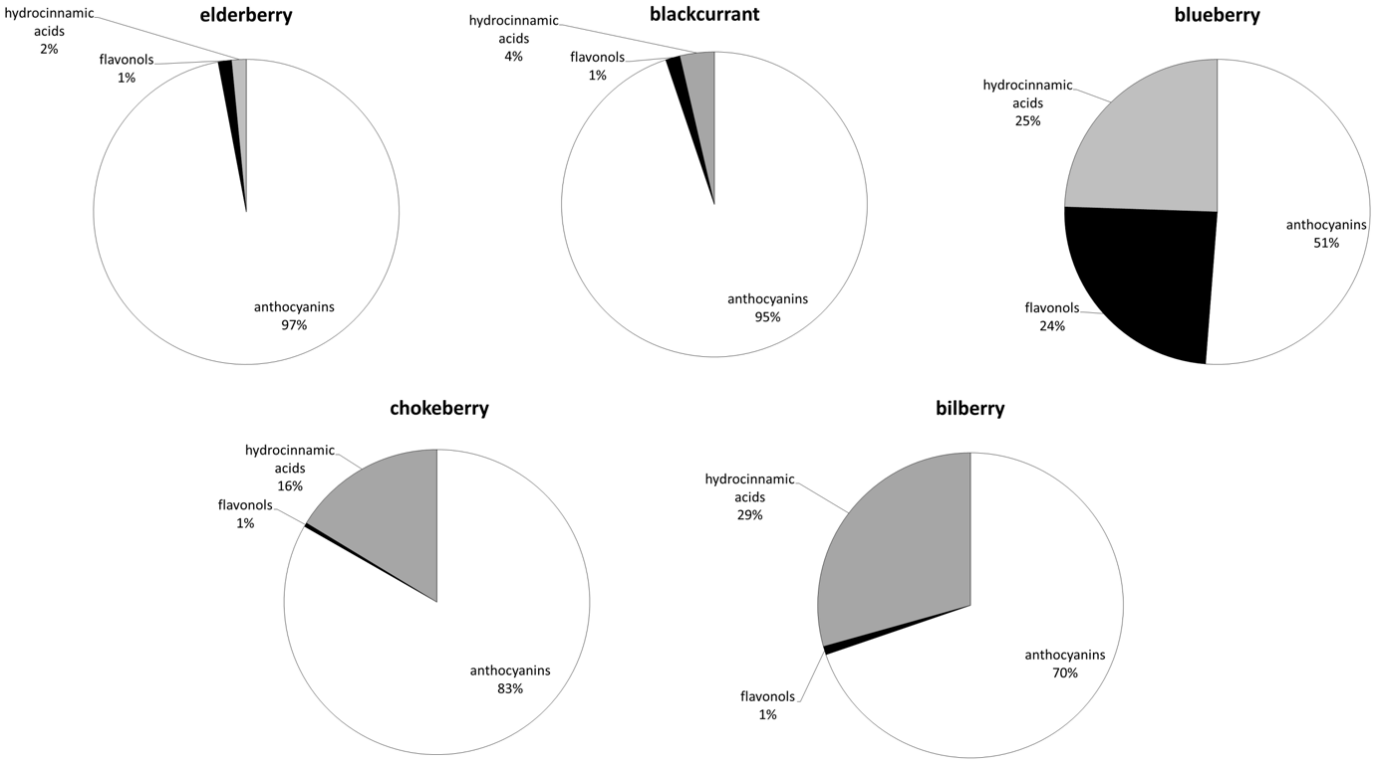
Distribution of total phenolic uptake according to particular phenolic groups.

Furthermore, the quantification of total phenolic uptake in the cells was achieved and analysed according to individual compounds (Figure 4). Results indicate, that in addition to the total content of phenolic compounds entering the cells a key factor determining the antioxidative action of berry juices was also the ratio between the compounds. Where high content levels of anthocyanins and very low content levels of flavonols and hydroxycinnamic acids were measured a lower intracellular oxidation has been detected. Specifically, intracellular oxidation increased with higher consumption of hydroxycinnamic acids and lower consumption of anthocyanins in the cells of *Saccharomyces cerevisiae.* Intracellular oxidation also increased when the consumption of analyzed phenols was rather low.

Which phenolic compounds, either alone or in synergism with others, contribute the most to biological effects of analyzed berries juice is still an open question. Anthocyanins are considered to be the most effective antioxidants [25] among the analysed phenolic compounds determined in the berries. The reason is twofold; firstly, high levels of anthocyanins are present in berry species and secondly, their transport across the membranes of different mammalian cells, (such as the blood-barrier) is rapid.

As demonstrated in some previous reports, the differences in antioxidant capacity could be ascribed to individual molecular structures of bioactive compounds. Some possible explanations are as follows: (a) the increasing number of hydroxyl groups may increase the antioxidant activity [3], [26]; (b) the o-dihydroxy structure in the B ring confers higher stability to the radical form and participates in electron delocalization [3], and thus, the dihydroxylation in the 3′,4′positions of the B ring plays an important role in antioxidant activity; (c) the glycosylation of flavonoids may reduce their activity when compared to that of the corresponding aglycones [27]; (d) the unsaturation in the C ring allows electron delocalization across the molecule for stabilization of aryloxy radicals due to existing conjugation; and (e) the 3- and 5-OH groups with 4-oxo function in the A and C rings are required for maximum radical scavenging potential [3].

Flavonols (quercetin, myricetin, kaempferol and others) possess high antioxidant activity as their structure allows a more effective antioxidant activity compared to the structure of anthocyanins. The quercetin 2,3 double bond in conjunction with a 4-oxo function in the C ring enables electron delocalization from the B ring, and showed extensive resonance, resulting in significant effectiveness for radical scavenging [3]. Quercetin has a structure similar to that of cyanidin in the A and B rings (3′,4′-dihydroxy substituents in the B ring and conjugation between the A and B rings) and the same number and arrangement of five hydroxyl groups. This suggested that quercetin may significantly contribute to antioxidant potential of natural products because its structure effectively satisfies the stabilization of the aryloxyl radical after hydrogen donation. An additional OH group at B ring 5′ position of quercetin, as well as in myricetin, increases the oxygen radical absorbance capacity.

Glycosylation of the anthocyanidins and quercetin may also modulate their antioxidant activity [28]. The preferred glycosylation site on the flavonoids is the 3 position and, less frequently, the 7 position. Glucose is the most common sugar residue, but others include galactose, rhamnose, and xylose [3]. Using quercetin as an example, 3-glycosylation in the C ring for glucose had the highest antioxidant activity *in vitro* compared to the others, and the order of antioxidant potency was usually 3-glucoside >3-rhamnoside >3-arabinoside ≈ 3-galactoside. The differences in antioxidant potency originate from the orientation, number, and distribution of hydroxyl group of the conjugated sugars.

## Conclusions

The contribution of individual phenolics to total antioxidant capacity was generally dependent on their structure and content levels in berry juices. The results of this study provide guidance as to the relative potential health benefits of different berries or berry juice. However, to understand the mechanisms of positive health effects after consumption of berries even better, there are still questions to be answered about bioavailability, metabolism, molecular targets, and bioactivity of phenolics in the human body.
